# Editorial: Meaningful participation and sensory processing

**DOI:** 10.3389/fpsyg.2022.1045650

**Published:** 2022-10-11

**Authors:** Antoine Bailliard, Winnie Dunn, Catana Brown, Batya Engel-Yeger

**Affiliations:** ^1^Department of Orthopaedic Surgery, Duke University, Durham, NC, United States; ^2^Department of Occupational Therapy, University of Missouri, Columbia, MO, United States; ^3^Department of Occupational Therapy, Midwestern University, Glendale, AZ, United States; ^4^Occupational Therapy Department, University of Haifa, Haifa, Israel

**Keywords:** sensory processing, participation, meaningful activities, everyday living activities, sensory modulation

Sensory processing has been studied across many scientific disciplines using different epistemologies and ontologies. The diversity in philosophical and scientific approaches has yielded different sensory processing theories with varying assumptions and conceptualizations of what sensory processing is and how it relates to health, cognition, the environment, and doing activities. It has also yielded different interpretations of the wide range of sensory processing patterns that exist among humans and their naturally occurring biological conditions (e.g., autism spectrum disorders, schizophrenia, post-traumatic stress disorder, traumatic brain injury, developmental disorders, etc.). Studies have shown that the fit between a person's sensory capacities and their sensory environment is associated with mental health, physical health, emotional regulation, and a person's capacity to perform tasks and activities. However, many studies have embraced a biomechanical model of sensory processing which reduces sensory processing to a mechanistic transmission of sense data from the environment through sense organs and along the central nervous system to be represented, integrated, and processed by the brain. Studies using a biomechanical model often employ sophisticated neural imaging or occur in heavily controlled labs designed to isolate sensations in a manner that is a contextual, ahistorical and does not reflect the lived sensory experiences of humans.

Although there have been significant advances in research methods across disciplines (e.g., neuroimaging in the medical sciences, non-representational methods in the social sciences), there remains a gap in the literature exploring the relationship of human sensory processing with doing meaningful activities, community integration, and inclusion in society. Research addressing this gap in the literature is necessary to inform interventions, policies, and other initiatives that support the inclusion and quality of life of all individuals regardless of their sensory processing capacities.

The purpose of this Research Topic, *Meaningful participation and sensory processing*, is to expand holistic understandings of the relationship of human sensory processing with participation in meaningful activities, inclusion, and community integration. This of this Research Topic of 14 research articles is an important contribution toward this endeavor and demonstrates the central role of sensory processing to meaningful participation in everyday activities, health, and wellbeing.

Three articles advance knowledge on the relationship of interoception and participation. Interoception, the ability to identify and respond to internal bodily stimuli, is situated as a central factor in the lived experience of everyday activities. Schmitt and Schoen present a new conceptualization of interoception as a complex multidimensional system consisting of a bidirectional interplay between the brain and the body to maintain homeostasis with an everchanging internal and external environment. They argue interoception is a foundation for meaningful participation and highlight the importance of pursuing evidence-based practices to address interoception in practice. Kalingel-Levi et al. contribute to this growing area of sensory research with a qualitative design that explores the experience of pain among autistic adults. Their findings highlight the critical role of awareness and communication in participants' experiences of pain and the profound impact those experiences have on their coping strategies, function, and participation. To enhance research and practice, Dunn et al. developed the Sensory Profile Interoception (SPI) scale to identify patterns of participation in activities that are associated with high and low levels of interoception (i.e., Interoceptive Impact). To demonstrate construct validity, Dunn et al. correlated the SPI with the Adolescent/Adult Sensory Profile, the Perth Alexithymia Scale, the Body Awareness Scale, and the State-Trait Anxiety Inventory. Their scale is an important contribution to developing evidence-based research and practice on interoception and its relationship to participation.

Further evidence of the centrality of sensory processing to everyday participation is provided by Wallisch et al. who explored the extent to which sensory processing affects children's attention to food cues. Their study used eye-tracking and sensory profiles to examine the relationship between oral sensory sensitivity and attentional bias to food among children. Results showed that children with high oral sensory sensitivity oriented more quickly to and spent more time looking at non-food logos than food logos.

Two articles highlighted the centrality of sensory processing to sleep, an essential activity that significantly affects mental and physical health. Hartman et al. found that sensory processing patterns affect the sleep of all children. In their study, children with sensory sensitivities experience more negative sleep behaviors than their counterparts. Lane et al. performed a scoping review to explore the relationship between sleep and sensory processing in autism. They found studies often report a relationship between sleep concerns and sensory reactivity differences; however, conclude that relationship between sleep and sensory processing is multidimensional and requires additional research.

Another Research Topic of articles provided clear examples of the impact of sensory processing on participation in community, school, and family activities. Bagatell et al. analyzed sensory profiles, interviews, and GPS tracking data with autistic adults to explore how their sensory processing patterns affect their community participation. Participants with patterns of sensory sensitivity and sensory avoiding reported spending less time in the community and visited fewer places because places felt overwhelming and fatiguing. Agostine et al. used a postcritical ethnography in two middle school classrooms and found that the students with multiple disabilities had few opportunities for rich sensory experiences and that their days are often filled with periods of waiting passively. Little et al. used a mixed methods analysis to examine how children's sensory response patterns are associated with caregiver strategies. Study findings demonstrate that caregivers employ strategies that are specific to their child's sensory response patterns and not related to diagnosis, mental age, or chronological age. Their study demonstrates the impact of sensory responsivity on caregiver activities throughout the day. Recognizing this important relationship, Ben-Sasson et al. validated a new pediatric Family Accommodation Scale for Sensory Over-Responsivity (FASENS) to measure the daily changes families make to accommodate a child. They found that typical families often accommodate their activities for children; however, families of children with health conditions enact more accommodations as evidence by higher scores on the FASENS. Daly et al. embraced a strength-based approach to understanding sensory processing and participation by using a meta-ethnography to explore the successful occupational experiences of family participation among families with autistic children. The study demonstrated the centrality of sensory experiences to family life and highlighted the importance of living with unpredictability for successful participation in family life.

Sensory processing has an undeniable impact on participation in meaningful activities that affect health and quality of life. May-Benson et al. examined the relationship of childhood sensory processing and related motor performance patterns and later quality of life as an adult. Their study found that sensory discrimination and modulation accounted for one-quarter of the variance in quality of life in adults.

Despite the importance of sensory processing to participation and health, the variability in sensory processing patterns across people is a challenge to research and there is a need to develop innovative methods. Clément et al. demonstrate the importance of using participatory methods to highlight the experiential knowledge of autistic children, youth, and adults to understand participation from their perspective. Their findings demonstrate how the use of innovative methods that allow autistic persons to speak of their bodily-sensing experiences on their own terms can lead to new and authentic ways of understanding participation that should be considered to reconceptualize the International Classification of Functioning (ICF).

Indeed, sensory processing patterns vary significantly among humans. Dean et al. analyzed sensory patterns from the Sensory Profile 2 across a national sample of children to investigate whether variations in sensory processing represent a natural variability or a problematic aspect of disability. Their analyses demonstrated that children in all groups exhibited different rates of certain sensory patterns thereby suggesting sensory differences cannot be associated with problematic behaviors.

Together, this Research Topic represents an important advancement in knowledge drawing a specific connection between sensory processing patterns and participation in meaningful activities. This Research Topic demonstrates that sensory processing patterns differ across groups of individuals and these differences have an impact on their participation in meaningful activities. Together, these articles demonstrate that sensory processing patterns have a central impact on health, quality of life, and participation in meaningful activities. More research is needed to deepen understandings of how sensory health (i.e., whether sensory capacities match sensory environments and the sensory demands of activities) affects participation, inclusion, community integration, and belonging.

## Author contributions

All authors listed have made a substantial, direct, and intellectual contribution to the work and approved it for publication.

## Conflict of interest

The authors declare that the research was conducted in the absence of any commercial or financial relationships that could be construed as a potential conflict of interest.

## Publisher's note

All claims expressed in this article are solely those of the authors and do not necessarily represent those of their affiliated organizations, or those of the publisher, the editors and the reviewers. Any product that may be evaluated in this article, or claim that may be made by its manufacturer, is not guaranteed or endorsed by the publisher.

